# On the Much‐Improved High‐Voltage Cycling Performance of LiCoO_2_ by Phase Alteration from O_3_ to O_2_ Structure

**DOI:** 10.1002/smsc.202400162

**Published:** 2024-08-01

**Authors:** Mingwei Zan, Hongsheng Xie, Sichen Jiao, Kai Jiang, Xuelong Wang, Ruijuan Xiao, Xiqian Yu, Hong Li, Xuejie Huang

**Affiliations:** ^1^ Beijing Frontier Research Center on Clean Energy Institute of Physics Chinese Academy of Sciences Beijing 100190 China; ^2^ Center of Materials Science and Optoelectronics Engineering University of Chinese Academy of Sciences Beijing 100049 China; ^3^ Institute of Physics Chinese Academy of Sciences Beijing 100190 China; ^4^ School of Physical Sciences University of Chinese Academy of Sciences Beijing 100049 China

**Keywords:** chemomechanics, high‐voltage performances, lithium cobalt oxides, lithium‐ion batteries, O_2_ phases

## Abstract

Lithium cobalt oxide (LiCoO_2_) is an irreplaceable cathode material for lithium‐ion batteries with high volumetric energy density. The prevailing O_3_ phase LiCoO_2_ adopts the ABCABC (A, B, and C stand for lattice sites in the close‐packed plane) stacking modes of close‐packed oxygen atoms. Currently, the focus of LiCoO_2_ development is application at high voltage (>4.55 V versus Li^+^/Li) to achieve a high specific capacity (>190 mAh g^−1^). However, cycled with a high cutoff voltage, O_3_–LiCoO_2_ suffers from rapid capacity decay. The causes of failure are mostly attributed to the irreversible transitions to H1‐3/O_1_ phase after deep delithiation and severe interfacial reactions with electrolytes. In addition to O_3_, LiCoO_2_ is also known to crystalize in an O_2_ phase with ABAC stacking. Since its discovery, little is known about the high‐voltage behavior of O_2_–LiCoO_2_. Herein, through systematic comparison between electrochemical performances of O_3_ and O_2_ LiCoO_2_ at high voltage, the significantly better stability of O_2_–LiCoO_2_ (>4.5 V) than that of O_3_–LiCoO_2_ in the same micro‐sized particle morphology is revealed. Combining various characterization techniques and multiscale simulation, the outstanding high‐voltage stability of O_2_–LiCoO_2_ is attributed to the high Li diffusivity and ideal mechanical properties. Uniform Li^+^ distribution and balanced internal stress loading may hold the key to improving the high‐voltage performance of LiCoO_2_.

## Graphical abstract

A systematic comparison between electrochemical performances of O_3_ and O_2_–LiCoO_2_ at high voltages is made and the significantly better stability of O_2_–LiCoO_2_ (>4.5 V) than O_3_–LiCoO_2_ is revealed. Comprehensive characterizations and multiscale simulations attribute the outstanding high‐voltage stability of O_2_–LiCoO_2_ to the high Li diffusivity and ideal mechanical properties.

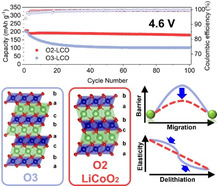

## 1 Introduction

In recent decades, high‐energy‐density lithium‐ion batteries (LIBs) have played a pivotal role in advancing electric vehicles, electronic devices, and large‐scale energy storage.^[^ ^1^, ^2^ ^]^ Developing LIBs with higher energy density remains a constant pursuit. Among the major components of LIBs, the cathode materials currently limit the improvement of energy density. Commercially available cathode materials mainly include layered materials such as LiCoO_2_ (LCO) and LiNi_*x*_Mn_*y*_Co_(1−*x*−*y*)_O_2_ (NMC) materials, spinel‐phase LiMn_2_O_4_ materials, and olivine‐phase LiFePO_4_ family.^[^ ^3^, ^4^ ^]^ Among them, LCO has the highest compacted density and, therefore, volumetric energy density. This characteristic secures its irreplaceable role in applications such as consumer electronics and other compact devices.^[^ ^5^, ^6^ ^]^

LCO crystalizes in various phases, among which the O_3_ phase dominates the market.^[^ ^7^ ^]^ The O_3_ nomenclature derives from Delmas's concept of layered materials classification.^[^ ^8^ ^]^ “O” represents Li–O_6_ or TM–O_6_ octahedra, and “3” signifies that the same octahedral layer is repeated every three layers within a unit cell. In layered materials, Li–O_2_ and TM–O_2_ layers stack alternately. The smallest repeating unit of O_3_–LCO, therefore, has six layers of octahedron. The oxygen atoms are arranged in a hexagonal close‐packed manner following the ABCABC (A, B, and C stand for lattice sites in the close‐packed plane) pattern. Common layered cathode materials, such as LiNiO_2_, LiNi_0.8_Co_0.1_Mn_0.1_O_2_, and Li_1+*x*_TM_1−*x*_O_2_, all share the same O_3_‐type structure.^[^ ^9^, ^10^, ^11^ ^]^ Since its discovery by Goodenough in 1980, the specific capacity of O_3_–LCO has been continuously increasing as the charging cutoff voltage rises.^[^ ^12^ ^]^ Currently, the reversible specific capacity of O_3_–LCO can reach 190 mAh g^−1^, corresponding to a voltage of ≈4.55 V (versus Li^+^/Li). Ongoing research is directed toward the development of O_3_–LCO with a cutoff voltage exceeding 4.6 V.^[^ ^13^, ^14^, ^15^ ^]^ However, the challenge is huge since high‐voltage O_3_–LCO faces serious degradation. For instance, after extensive lithium deintercalation (Li content <0.3), the O_3_ structure undergoes slip between octahedral layers, leading to the formation of the H1‐3 and subsequent O_1_ phases, accompanied by a significant change in the unit cell volume.^[^ ^16^, ^17^, ^18^ ^]^ LCO particles are unable to withstand such large volume changes, resulting in crack formation and surface exposure.^[^ ^19^, ^20^, ^21^ ^]^ Moreover, the surface reactions involving Co^4+^ with the electrolyte are aggravated under high‐voltage conditions.^[^ ^22^, ^23^ ^]^ Nevertheless, oxygen becomes active in charge transfer at high voltages, leading to the potential loss of lattice oxygen and the formation of nonconductive Co_3_O_4_ on the surface, causing rapid capacity decay.^[^ ^24^, ^25^, ^26^ ^]^

In addition to O_3_ stacking, LiCoO_2_ also has an isostructural polymorph with O_2_ stacking. The coordination of Li–O_6_ and Co–O_6_ remains the same, but oxygen atoms stack following the ABAC pattern, which leads to a repetition of CoO_6_ layer twice within the smallest unit cell. O_2_–LCO is a thermodynamically metastable phase. Since high‐temperature sintering of lithium and cobalt precursors typically yields the O_3_ phase, O_2_–LCO is usually obtained through ion exchange from the P_2_‐type Na_0.7_CoO_2_ (P_2_–NCO).^[^ ^27^ ^]^ In 1982, following the discovery of O_3_–LCO by Goodenough,^[^ ^12^ ^]^ Delmas and colleagues first synthesized O_2_–LCO via ion exchange.^[^ ^28^ ^]^ In 2000, J. R. Dahn and others conducted a study on the phase transitions and thermal stability of O_2_–LCO during charge and discharge.^[^ ^29^ ^]^ They found that O_2_–LCO exhibited a series of reversible phase transitions and comparable thermal stability to O_3_–LCO. In O_3_–LCO, several phase transitions occur during delithiation accompanying the layer sliding. According to the detailed calculation by Ceder et al.^[^ ^30^ ^]^ O_3_–LCO would first transit into H1‐3 phase at about *x* = 0.25, followed by the transition to O_1_ phase at about *x* = 0.16. The detailed phase transition process of delithiated O_2_–LCO (0.16 < *x* < 1) was revealed by Delmas and collaborators in 2002,^[^ ^31^ ^]^ showing that O_2_–LCO goes through O_2_, T_2_, O_6_, O_2_', and O_2_ upon delithiation.^[^ ^31^ ^]^ Over the years, for O_2_–LCO, most reports mainly focused on the phase transition while overlooking its electrochemical performance to some extent.^[^ ^32^, ^33^ ^]^ Though O_3_– and O_2_–LCO share similar voltage profiles, O_2_–LCO can transfer back to O_2_ stacking when deeply delithiated instead of O_1_ stacking as in O_3_–LCO. It may hold the potential to outperform O_3_–LCO at high voltage. A systematic comparison between O_3_–LCO and O_2_–LCO during cycling at high voltages is necessary.

In this study, plate‐like O_2_–LCO particles were synthesized through an ion‐exchange experiment and O_3_–LCO particles with identical morphology were obtained through heat treatment. A comprehensive comparison of the electrochemical cycling performance between O_3_– and O_2_–LCO at high cutoff voltages (>4.5 V) was conducted. Results demonstrate a much‐improved high‐voltage cycling stability of LCO by switching from O_3_ to O_2_ phase. Combining thorough characterization and simulation, we propose a chemomechanical failure mechanism for LCO particles during high‐voltage cycling.

## 2 Results and Discussion

### 2.1 Structure

There were several recipes for O_2_–LCO synthesis reported in the past. Delmas et al. first used a methanol solution containing LiCl to convert P_2_–NCO into O_2_–LCO after a 3‐day reaction.^[^ ^27^ ^]^ This method has a long reaction time and leaves behind a significant amount of P2 impurity. Later, J. R. Dahn and others improved the method by using water with lithium salts like LiCl and LiNO_3_ as agents.^[^ ^34^ ^]^ In addition, the combination of hexanol and LiBr is also feasible.^[^ ^29^ ^]^ According to the early literature on O_2_–LCO and recent literatures on O_2_‐type lithium‐rich materials, the condition of ion‐exchange has a large impact on the final product's morphology, structure, and electrochemical performance.^[^ ^34^, ^35^ ^]^ Taking into account of reaction time and product purity, the aqueous‐solution‐based method was chosen here.

**Figure 1:**
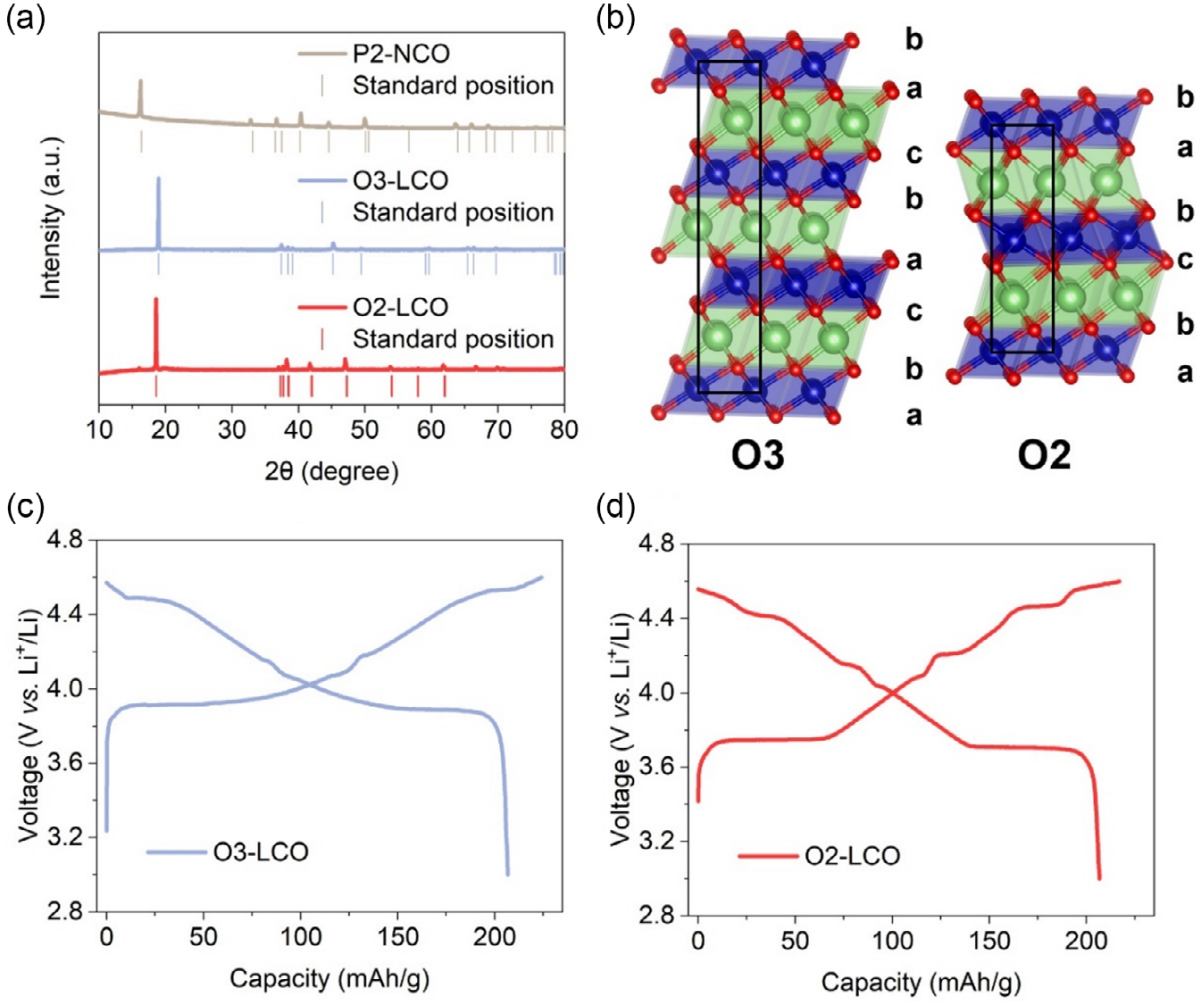
a) The measured XRD patterns of P_2_–NCO, O_3_–LCO, and O_2_–LCO. b) The structural illustration of O_3_ and O_2_ stacking. c,d) The first‐cycle voltage profiles of O_3_– and O_2_–LCO between 3 and 4.6 V (versus Li^+^/Li), respectively.

Figure S1a,b, Supporting Information, shows the scanning electron microscope (SEM) images of synthesized P_2_–NCO precursor. P_2_–NCO particles exhibit plate‐like morphologies and aggregate together. The larger particles are decorated with numerous small dense particle protrusions on their surface. From the X‐ray diffraction (XRD) results (**Figure** **1**a), P_2_–NCO sample exhibits diffraction peaks of the standard P6_3_/mmc space group which indicates phase purity. SEM images of O_2_–LCO sample after ion exchange are shown in Figure S1c,d, Supporting Information. The plate‐like particle morphology is largely inherited from P_2_–NCO. Differences can be found in that the surface of O_2_–LCO particles is smooth without any decorations and the particles are relatively dispersed instead of aggregation. O_2_–LCO is a thermodynamically metastable phase that transforms into the O_3_ phase at high temperatures. This process involves partial Co—O bond breaking but has minimal impact on the particle morphology.^[^ ^27^ ^]^ Plate‐like O_2_–LCO particles are then heated at 600 °C and transformed into O_3_–LCO (Figure S1e,f, Supporting Information). O_3_–LCO particles obtained this way share the identical size and morphology with the O_2_–LCO sample, which can eliminate the influence of morphology and particle size on the electrochemical performance difference between O_3_– and O_2_–LCO.

Figure 1b illustrates the O_3_ and O_2_ stacking pattern, and Figure 1c,d presents the typical electrochemical curve of O_3_– and O_2_–LCO (3–4.6 V versus Li^+^/Li). The charge and discharge curves measured here are similar to those reported in the literature.^[^ ^7^, ^29^, ^31^ ^]^ O_2_–LCO exhibits a pronounced voltage plateau at 3.75 V during the initial charging, which is lower than the 3.92 V voltage plateau of O_3_–LCO. O_3_–LCO also shows two phase‐transition plateaus at 4.25 and 4.55 V during charging. Detailed discussions on structural phase transitions during charge and discharge processes will be provided in the in situ XRD experiments. It should be noted that the voltage curve presented here for O_2_–LCO is that of the second cycle. The first cycle is shown in Figure S2, Supporting Information. During the first cycle, O_2_–LCO experiences an abnormal voltage increase after the initial lithiation to Li1, which will disappear only after the charging voltage exceeds 4.4 V. The subsequent first cycle discharge curve of O_2_–LCO is not affected. A deeper look at the XRD of O_2_–LCO reveals a small extra peak at 16.05° aside from other peaks matching well with the standard ones of P6_3_/mmc space group, implying a trace amount of P_2_–NCO impurity as the 16.05° peak matches its (002) peak position. Despite conducting ion exchange for up to 24 h, a complete Li–Na exchange cannot be obtained, which is consistent with previous reports.^[^ ^27^, ^29^ ^]^ In addition, during the ion‐exchange process, slip between octahedral layers occurs creating stacking faults, which may also contribute to the impurity peak.^[^ ^36^, ^37^ ^]^

### 2.2 Electrochemical Performance

The first discharge specific capacity of synthesized O_3_– and O_2_–LCO at 4.6 V is 206.8 and 213.9 mAh g^−1^, respectively, slightly lower than that of commonly reported O_3_–LCO,^[^ ^15^, ^38^, ^39^ ^]^ which is very likely a consequence of the trace amount of impurity. However, this does not affect the focus of the current comparative study. From Figure 1d, it can be seen that after reaching a charging capacity of 65 mAh g^−1^, the voltage curve of O_2_–LCO starts to rise and becomes very similar to that of O_3_–LCO. We also tested the charge and discharge capacities of other O_3_–LCO at high voltages, as shown in Figure S2, Supporting Information. The voltage curves above 4.2 V for O_3_– and O_2_–LCO are quite consistent (see the dashed ellipse in Figure S2c, Supporting Information), which indicates that at all cutoff voltages exceeding 4.5 V, O_2_–LCO can deliver a capacity similar to that of O_3_–LCO, albeit with slightly lower discharge specific energy. Excluding the influence of the impurity phase in the current sample, the actual pure O_2_–LCO should deliver a discharge capacity almost equivalent to conventional O_3_–LCO at high cutoff voltages.

**Figure 2:**
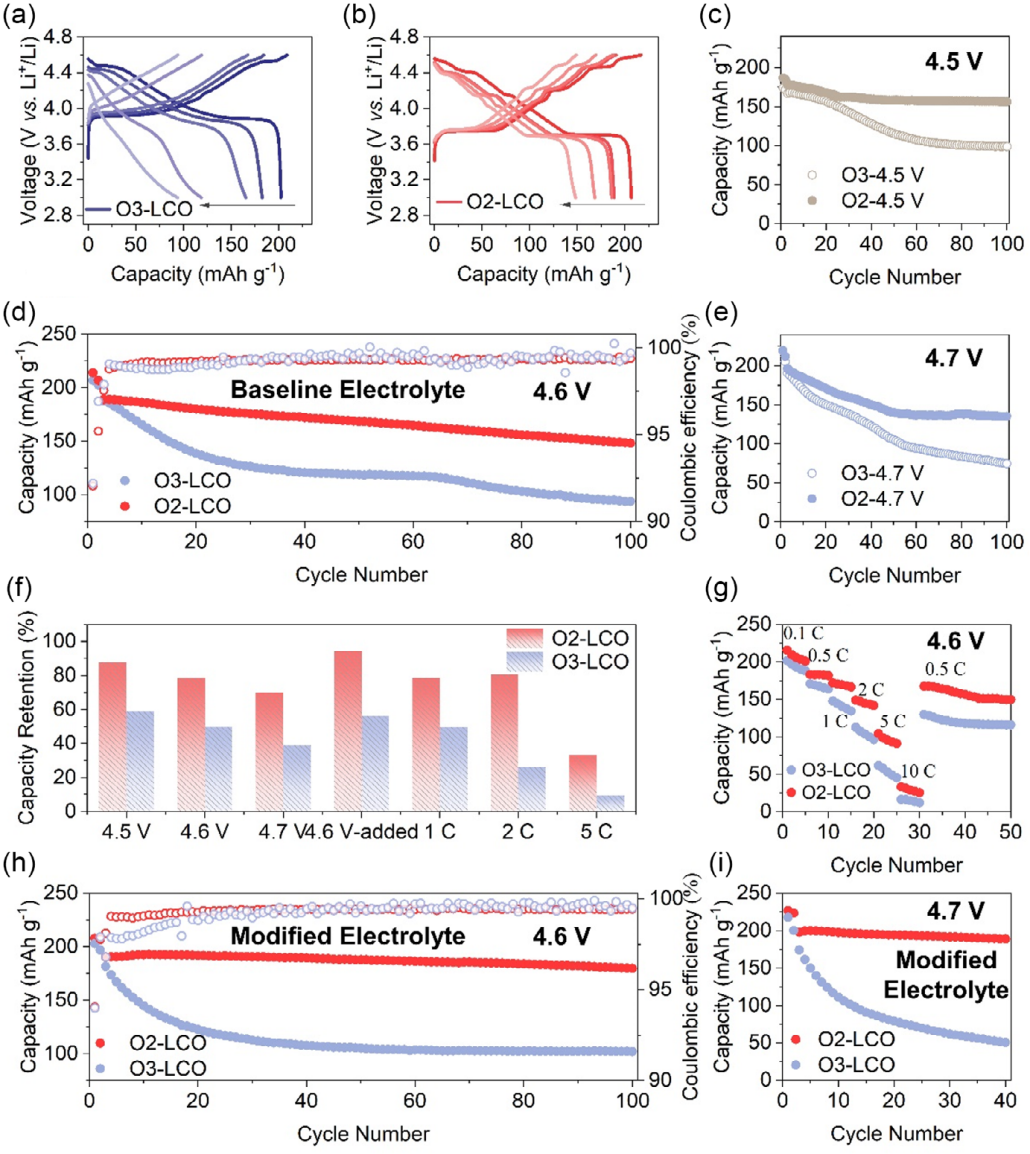
Cycling performance comparison between O_3_– and O_2_–LCO. a,b) The typical voltage profile evolution of O_3_– and O_2_–LCO during prolonged cycling between 3 and 4.6 V versus Li^+^/Li with a baseline electrolyte. c–e) The cycling capacity evolution of O_3_– and O_2_–LCO with charge cutoff voltage of 4.5, 4.6, and 4.7 V, respectively (0.1C for the first two cycles and 0.5C for the rest of cycles, 1C = 200 mA g^−1^). f) The capacity retention difference between O_3_– and O_2_–LCO after 100 cycles under different cutoff voltages and at different cycling rates (4.6 V added indicates the electrolyte with additives). g) The rate‐compatibility difference between O_3_– and O_2_–LCO. h,i) Cycling capacity evolution of O_3_– and O_2_–LCO with a high‐voltage‐optimized electrolyte under 4.6 and 4.7 V cutoff voltages, respectively (0.1 C for the first two cycles and 0.5 C for the rest of cycles, 1 C = 200 mA g^−1^).

The long‐term electrochemical performance of O_3_– and O_2_–LCO at different cutoff voltages is depicted in **Figure** **2**. Figure 2a,b displays the charge–discharge curves for O_3_– and O_2_–LCO at 4.6 V cutoff voltage after the 1st, 5th, 10th, 50th, and 100th cycles. It is evident that O_2_–LCO maintains a certain degree of similarity in its electrochemical curve to the first cycle throughout the cycling process, with the phase‐transition plateau preserving after 100 cycles, indicating some structural reversibility during extended cycling. In contrast, O_3_–LCO not only exhibits substantial direct current polarization after 50 cycles but also experiences degradation of the electrochemical curve into a sloping line. Its capacity significantly decreases from the initial 206.8–93.7 mAh g^−1^, resulting in a capacity retention of only 49.9% after 100 cycles. In contrast, O_2_–LCO displays a high capacity retention of 78.4% (148.1 mAh g^−1^) after 100 cycles (Figure 2d). Additionally, O_2_–LCO maintains stable Coulombic efficiency during long cycling at 4.6 V, while O_3_–LCO exhibits more significant fluctuations. The much higher capacity retention rate of O_2_–LCO also leads to a better energy density retention even though O_2_–LCO shows slightly lower voltage than O_3_–LCO near the end of discharge (Figure S3, Supporting Information). Notably, the superior cycling stability of O_2_–LCO is observed not only at 4.6 V but also at 4.5 and 4.7 V cutoff voltages (Figure 2c,e) (0.1C for the first two cycles and 0.5C for the rest cycles, 1C = 200 mA g^−1^). Particularly at 4.7 V, O_2_–LCO only experiences a rapid capacity decay in the first 40 cycles and maintains the capacity without any noticeable loss after that (Figure 2e). The initial fast decay may be related to oxygen activation and the stable cycling after that still demonstrates the superior resilience of O_2_–LCO compared to O_3_–LCO. The excellent electrochemical stability of O_2_–LCO at high voltages above 4.5 V is somewhat unexpected, especially when the tested sample here does not have any surface modification and the electrolytes used here are simply commercial ones without any additives. Furthermore, O_2_–LCO is just an isostructural polymorph to O_3_–LCO where the only difference is the stacking pattern. They share similar chemical properties and go through the same redox processes during charge–discharge, which rules out the chemical cause for the evident performance difference. Thermodynamically speaking, O_2_–LCO is less stable than O_3_–LCO, which has the opposite trend against the observed electrochemical stability.

In addition to better high voltage compatibility, O_2_–LCO sample also exhibits better rate capability. As shown in Figure 2f,g, the capacities of O_2_–LCO at 1C, 2C, 5C, and 10C are 172.1, 149.1, 104.6, and 33.3 mAh g^−1^, respectively, while the corresponding capacities of O_3_–LCO are 147.7, 112.8, 61.9, and 16.4 mAh g^−1^. More surprisingly, after 100 cycles at 2C and 4.6 V, O_2_–LCO still retains a capacity of 142.1 mAh g^−1^, far exceeding the 45.3 mAh g^−1^ of O_3_–LCO (Figure 2f). Since the particle morphologies of LiCoO_2_ sample are identical, the improved rate performance of O_2_–LCO suggests better Li^+^ transport kinetics. To minimize the influence of interfacial reaction with electrolyte at high voltage, we further adopted some high‐voltage electrolyte additives and tested the 4.6 V cycling performance again. After 100 cycles, O_2_–LCO still maintained a much higher capacity retention rate (94.4%) than O_3_–LCO (56.2%). At the same undoped and uncoated conditions, O_2_–LCO appears to be more tolerant to high voltages than O_3_–LCO. From the perspective of capacity, O_2_–LCO has the potential to rival O_3_–LCO in high‐voltage applications.

### 2.3 Phase Transition and Li^+^ Transportation

**Figure 3:**
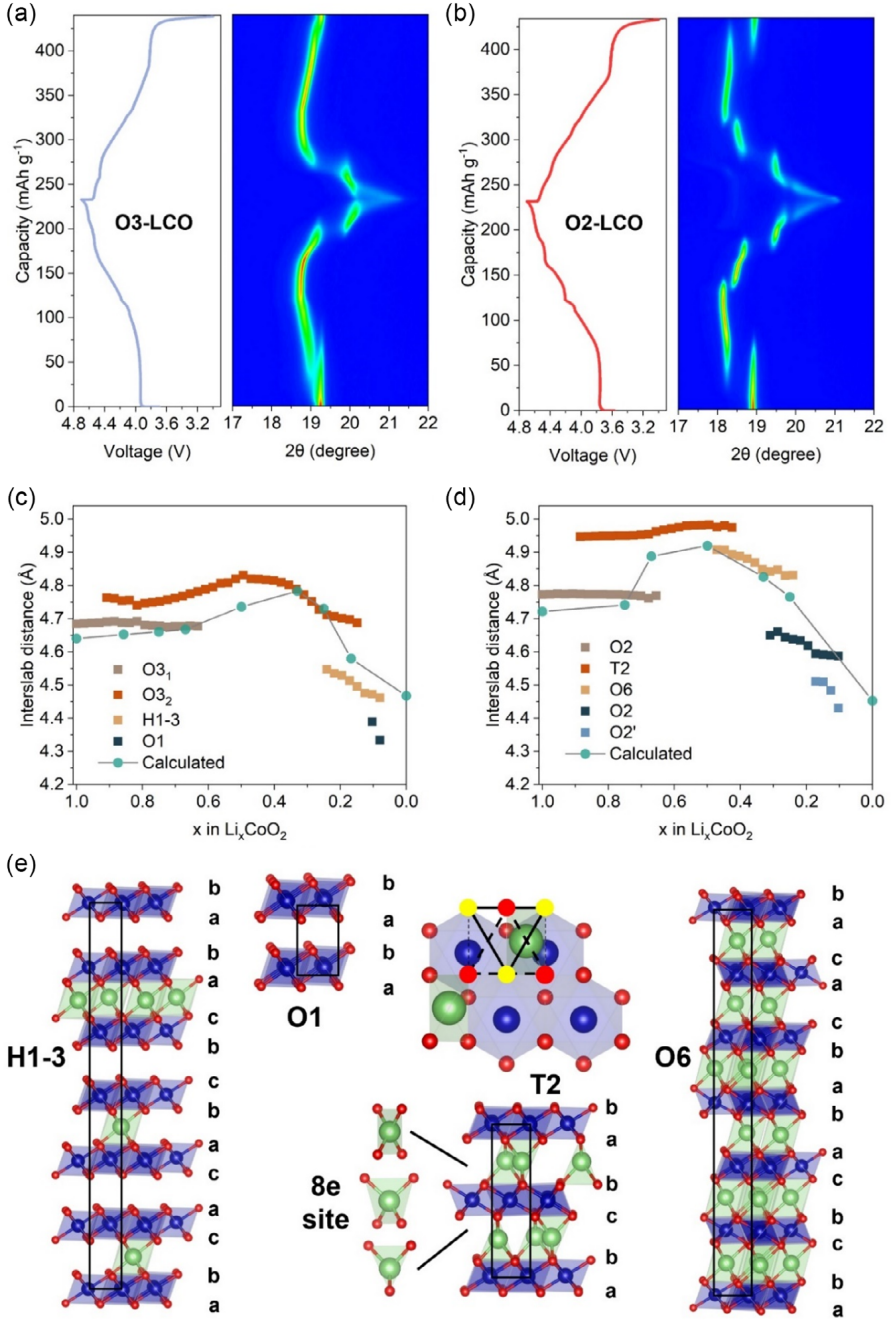
a,b) Electrochemical in situ XRD results for O_3_– and O_2_–LCO, respectively. c,d) The evolution of inter‐slab distance during delithiation, refined from in situ XRD data and from DFT calculation for O_3_– and O_2_–LCO, respectively. e) Structural illustration of different stacking phases that appear during delithiation of O_3_– and O_2_–LCO.

An in situ XRD experiment was conducted between 3 and 4.7 V versus Li^+^/Li to monitor the structural change of O_3_– and O_2_–LCO upon delithiation. Results are shown in **Figure** **3**a,b where only the part between 2θ = 17° and 22° is zoomed in to highlight the (003) peak position shift. O_3_–LCO shows three distinct phase transitions and four phases, while O_2_–LCO shows four distinct phase transitions and five phases. The overall trend of the inter‐slab distance change along the *c* axis is the same for both materials; it first increases and then rapidly decreases after 50% state of charge (SOC) (Figure 3c,d and S4, Supporting Information). Current results are consistent with previous reports in the literature.^[^ ^16^, ^18^ ^]^ It is worth noting that for O_2_–LCO, due to the increased repulsion brought by face sharing between Li–O_6_ and Co–O_6_ octahedron, the layer structure undergoes slip after a small portion of lithium is extracted, forming a new phase known as the T2 phase.^[^ ^31^, ^33^ ^]^ Li^+^ occupies the distorted tetrahedral 8e position, which causes the inter‐slab distance to suddenly increase from 4.7 to 4.95 Å around 25% SOC. Though large lattice parameter changes are usually considered unfavorable for structural integrity, later results would demonstrate that the emergence of T2 phase actually benefits the structural reversibility of O_2_–LCO. The O_2_–T_2_ phase transition has a smaller layer slip distance than that of O_3_–O_1_ transition, which soothes the lattice distortion during phase transition. Li^+^ occupying the tetrahedral sites is energetically more favorable than octahedral sites which helps stabilizing the T2 stacking. Furthermore, tetrahedral Li^+^ bonds stronger with O due to the lowered Li–O coordination number which helps hold the adjacent layers together. Density‐functional theory (DFT)‐based calculations were also conducted to evaluate the lattice parameter change upon delithiation. Table S1 and S2, Supporting Information, summarize the calculated lattice parameters at different SOC of LCO. For both O_3_– and O_2_–LCO, it can be seen that the lithium content has little effect on the lattice parameters in the *ab* plane but has a significant effect on the parameter along the *c* axis. Such results are consistent with many previous reports and are reasonable since delithiation from the Li–O_6_ layer mainly affects the inter‐slab interactions and, therefore, the lattice parameter along the *c* axis. Overall calculated inter‐slab distance is in great agreement with the refined results from in situ XRD measurement demonstrating the accuracy of the current DFT calculation.

**Figure 4:**
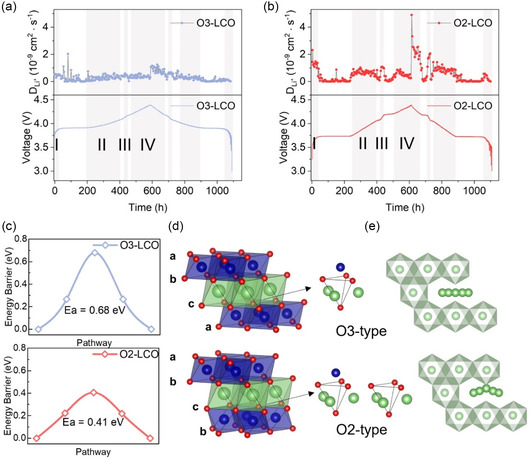
a,b) Total diffusivity of Li^+^ fitted from GITT experiment results for O_3_– and O_2_–LCO, respectively, throughout the whole charge–discharge cycle. Regions corresponding to solid‐solution processes are marked in grey. c) Energy barrier comparison between O_3_– and O_2_–LCO for a single Li^+^ vacancy migration estimated by NEB calculation. d) Illustration of the atomic environment difference between Li^+^ migration transition states in O_3_ and O_2_ stacking. e) Comparison of Li^+^ migration trajectories in O_3_ and O_2_ stacking projected onto *x–y* plane.

The inter‐slab distance has a decisive effect on the Li^+^ transportation. As Ceder et al. pointed out before,^[^ ^40^ ^]^ in layered cathode material, the tetrahedron height at the transition site along Li^+^ migration pathway, which equals the inter‐slab distance, is strongly correlated to the migration barrier. Because of the observed better rate capability of O_2_–LCO in Figure 2, the transport properties of Li^+^ in O_3_– and O_2_–LCO need careful examination. Galvanostatic intermittent titration technique (GITT) was adopted to estimate the Li^+^ diffusivities, as shown in **Figure** **4**a,b. The apparent Li^+^ diffusivity is fitted using the electrode cross‐sectional area instead of the electrode effective area since current O_3_– and O_2_–LCO samples share the same particle morphology and distribution. GITT‐based diffusivity estimation is achieved based on the assumption of a single‐phase solid‐solution reaction. Therefore, the voltage profile is purposefully divided into different solid‐solution regions and the average diffusivity for each region is listed in **Table** **1**. The results show that in all regions the diffusivity of Li^+^ in O_2_–LCO is more than twice that in O_3_–LCO, indicating overall better diffusion kinetics of Li^+^ in O_2_–LCO. Such measurement is also supported by DFT‐based nudged elastic band (NEB) calculations of the Li^+^ migration barrier. As shown in Figure 4c, the migration barrier for Li^+^ in O_2_–LCO is 0.41 eV, much lower than the 0.68 eV in O_3_–LCO. Li^+^ migration in LiCoO_2_ occurs through transitional tetrahedral sites. The stacking arrangement in O_3_–LCO ensures that each tetrahedral site shares faces with an adjacent Co atom in the CoO_6_ layer. Meanwhile, the O_2_ stacking leads to half of the tetrahedral sites sharing faces with no Co atom (Figure 4d). Therefore, the crystal configuration of O_2_–LCO reduces the repulsion experienced by Li^+^ during migration, accelerating the diffusion. Furthermore, as Figure 3c,d shows, throughout the whole delithiation process, O_2_–LCO has a larger inter‐slab distance than O_3_–LCO at almost any SOC, which would also lower the repulsion felt by Li^+^ during migration. Combining GITT test and calculation results, particular atomic arrangement and larger inter‐slab distance in O_2_–LCO enable smaller Li^+^ diffusion barriers and larger apparent diffusivity during charge–discharge, resulting in better rate capability. Faster Li^+^ diffusion can also make the spatial distribution of Li^+^ inside the particle more uniform during delithiation and probably smooth the phase‐transition process, which may benefit the cycling stability.

**Table 1:** Fitted Li+ diffusion coefficient (cm^2^ s^−1^).

Solid solution region	I	II	III	IV
O_2_	1.4 × 10^−9^	6.6 × 10^−10^	9.3 × 10^−10^	5.2 × 10^−10^
O_3_	4 × 10^−10^	3.1 × 10^−10^	4.6 × 10^−10^	3.7 × 10^−10^

### 2.4 Performance Degradation Mechanisms

**Figure 5:**
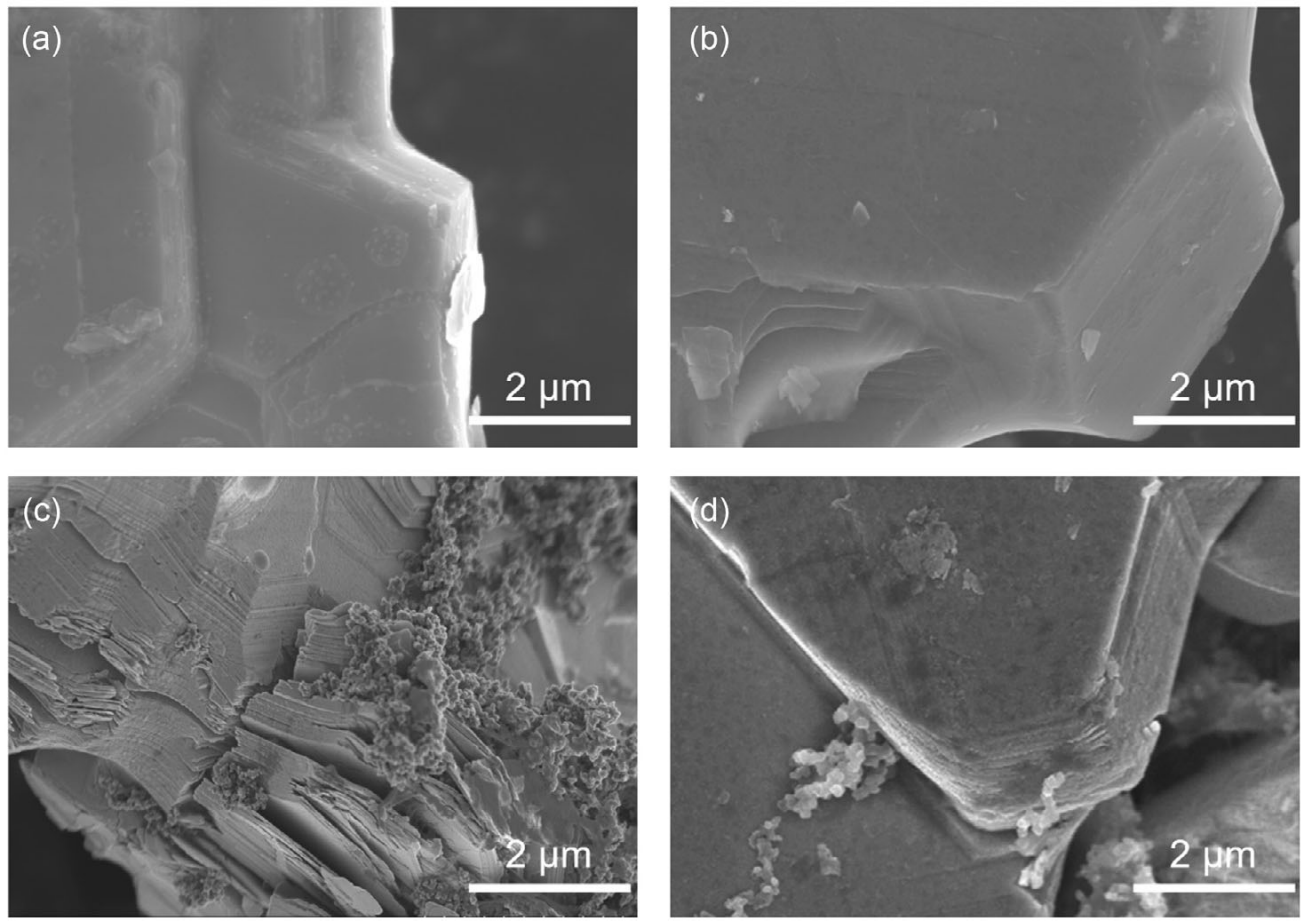
SEM images of a) pristine O_3_–LCO particle, b) pristine O_2_–LCO particle, c) cycled O_3_–LCO particle, and d) cycled O_2_–LCO particle. 100 cycles was conducted between 3 and 4.6 V versus Li^+^/Li at 0.5C with high‐voltage‐optimized electrolyte.

Let's take a step further. As the in situ XRD results suggest, for both O_3_– and O_2_–LCO, the unit cell goes through large volume variation as Li content changes, mostly caused by evident cell size variation along *c* axis. Nonuniform spatial distribution of Li^+^ in the particle would, therefore, lead to large internal strain, which could be detrimental to particle integrity upon long‐term cycling. **Figure** **5** displays the particle morphological change after 100 cycles at 0.5C between 3 and 4.6 V (versus Li^+^/Li) for O_3_– and O_2_–LCO. Before cycling, as seen in Figure 5a,b, SEM images zoomed in on the plate edge show no cracks either in‐plane or inter‐plane for both samples. After cycling, SEM image in Figure 5c for O_3_–LCO shows obvious disintegration of the particle. Thinner plates are exfoliating from the original plate and cracks are emerging from the plate. In contrast, for O_2_–LCO as shown in Figure 5d, the particle stays almost intact after long cycling with barely any cracks noticeable. Such observation verifies the aforementioned assumption to some extent. However, whether the particle morphological degradation is caused solely by nonuniform Li^+^ distribution needs further investigation.

**Figure 6:**
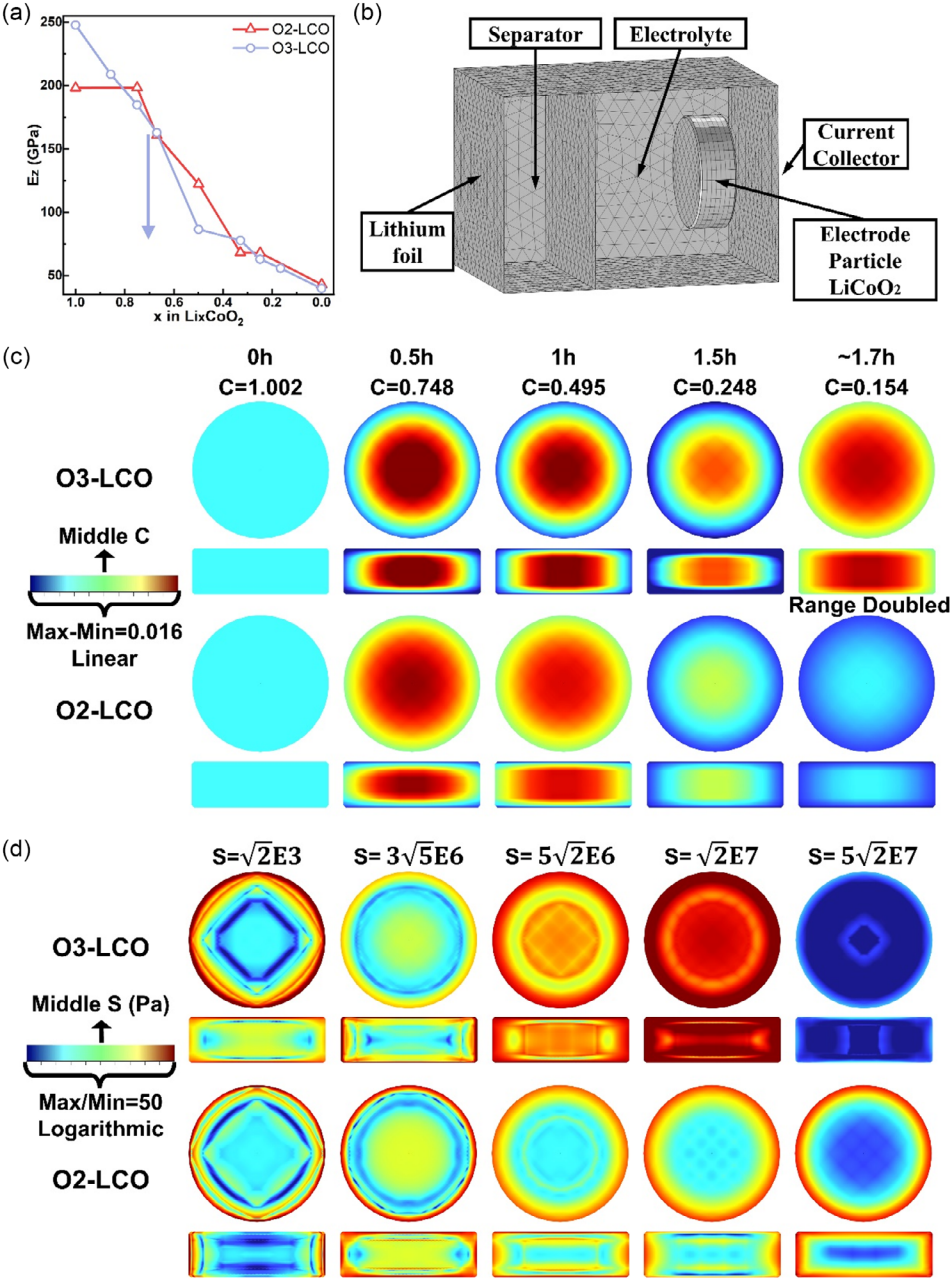
a) The variation of DFT‐calculated Young's modulus along *z* axis ([003] direction) for O_3_– and O_2_–LCO during delithiation. b) Illustration of single‐particle cell model used in the FEM simulation of a charge–discharge cycle at 0.5C. c) Simulated Li concentration distribution in the LCO particle at different SOC. d) Calculated von Mises stress distribution in the LCO particle at different SOC.

Though nonuniform Li^+^ distribution would inevitably build up strain in LCO, strain alone cannot tear the particle apart. In general, if the material is highly elastic, even with large internal strain a particle can still maintain morphological integrity by achieving a balance of internal stress. Therefore, to relate the Li concentration (strain) distribution with stress distribution, the elasticity of LCO material and its change upon delithiation need to be evaluated first. DFT‐based calculations of the elastic modulus for O_3_– and O_2_–LCO were carried out at different lithium content. Adopting the Voigt notation to represent the Young's modulus matrix of the material (detail in Experimental Section), we display the c33 element (elastic moduli along *z* direction, *E*_*z*_) as a function of Li content in O_3_– and O_2_–LCO in **Figure** **6**a. Overall, the elasticity of both LCO materials shows strong anisotropy (Table S3 and S4, Supporting Information), where the Young's moduli along the slab (*ab* plane) are very different from the one direct to the slab (*c* axis). This can be easily understood since in‐slab atoms are connected through covalent Co—O bonds while inter‐slab atoms are mostly connected through ionic Li—O bonds and weak van der Waals interactions. Upon delithiation, the quantity of Li—O bonds decreases, weakening the inter‐slab interaction. As a result, *E*_*z*_ gets monotonically smaller as Li content decreases (Figure 6a). Meanwhile, the Young's moduli along the *ab* plane (*E*_*x*_) stay almost the same (Table S3 and S4, Supporting Information). Smaller elastic moduli mean lower stress generation ability to counterbalance the stress loading. In other words, both O_3_– and O_2_–LCO become less resilient to *c*‐axis stress upon delithiation and more prone to exfoliate. However, the detailed evolution of *E*_*z*_ during Li extraction is different between O_3_– and O_2_–LCO. As marked in Figure 6a, a sudden drop of *E*_*z*_ happens when Li content reaches 0.5 in O_3_–LCO while for O_2_–LCO the decrement of *E*_*z*_ happens more continuously from Li0.66 to Li0.33. This difference may be related to the occurrence of T2 phase at about Li0.5 in O_2_–LCO, where Li^+^ prefers twisted tetrahedral sites bonding tighter with O. Strong Li—O bonds sustain the inter‐slab interaction and avoid the sudden softening of LCO material along *c* axis when Li content is below a certain level.

To examine the internal stress evolution upon delithiation, we constructed a half‐cell model with a plate‐like cylinder LCO particle as cathode (Figure 6b) and conducted finite‐element method (FEM)‐based electrochemical simulations (details in Experimental Section). Parameterized diffusivity, lattice strain, and elastic modulus were implemented. At 0.5C, the simulation results can well reproduce the measured charge–discharge voltage profile (Figure S6, Supporting Information). The distribution of Li concentration and internal stress from simulation results are displayed in Figure 6c,d for O_3_– and O_2_–LCO at representative SOC. And, 2D views along the plate and directional to the plate are sliced through the particle center. Upon charging (delithiation), there is a clear nonuniform distribution of Li^+^ in both O_3_– and O_2_–LCO particles, where the center region has a higher concentration, and the edge region has a lower concentration. Such distribution is reasonable as the outward Li^+^ flow is driven by the concentration gradient. However, the Li^+^ concentration gradient is much larger in O_3_–LCO than in O_2_–LCO during the whole delithiation process, which should be attributed to the lower diffusivity in O_3_ phase. At the same charging rate, a larger concentration is required to sustain the Li^+^ flow needed for O_3_–LCO. A larger concentration gradient leads to larger internal strain since the lattice parameter varies much from the particle center to the edge. For example, at around 50% SOC, the Li^+^ concentration at the central part of O_3_–LCO particle is still bigger than Li0.5 while it already gets below Li0.5 in the edge region. Large strain exists throughout the particle. Taking the sudden elasticity change around Li0.5 into account, one would expect strong and unbalanced internal stress to be generated in O_3_–LCO. The simulated stress distribution verifies this presumption. As shown in Figure 6d, internal stress is in general larger in O_3_–LCO particles compared to O_2_–LCO, especially around Li0.5 and Li0.25 SOC.

To further reveal whether slow Li^+^ diffusion or discontinuous elasticity change causes the huge internal stress in O_3_–LCO particles, we twitched the simulation parameter for O_2_–LCO and examined the results response. Figure S7, Supporting Information, displays the results if the Li^+^ diffusivity in O_2_–LCO is set to the value in O_3_–LCO. The resultant Li^+^ concentration distribution is almost the same as that in O_3_–LCO (Figure 6c), indicating the high sensitivity of it to the diffusivity change. However, since the elastic moduli of O_2_–LCO change continuously as Li content decreases, the internal stress does not rise to the level observed for O_3_–LCO (Figure 6d). In Figure S8, Supporting Information, the internal stress distribution is shown for O_2_–LCO when *E*_*z*_ profile is set the same as O_3_–LCO but diffusivity is untouched. Results show some increment of internal stress, especially around 50% SOC but not high enough comparable to that observed in O_3_–LCO (Figure 6d). Fast Li^+^ diffusion in O_2_–LCO leads to a more uniform distribution of Li concentration and smaller local strain. Summing up these results, it should be concluded that the large internal stress in O_3_–LCO during delithiation is a synergistic consequence of slow Li^+^ diffusion and discontinuous elasticity change.

**Figure 7:**
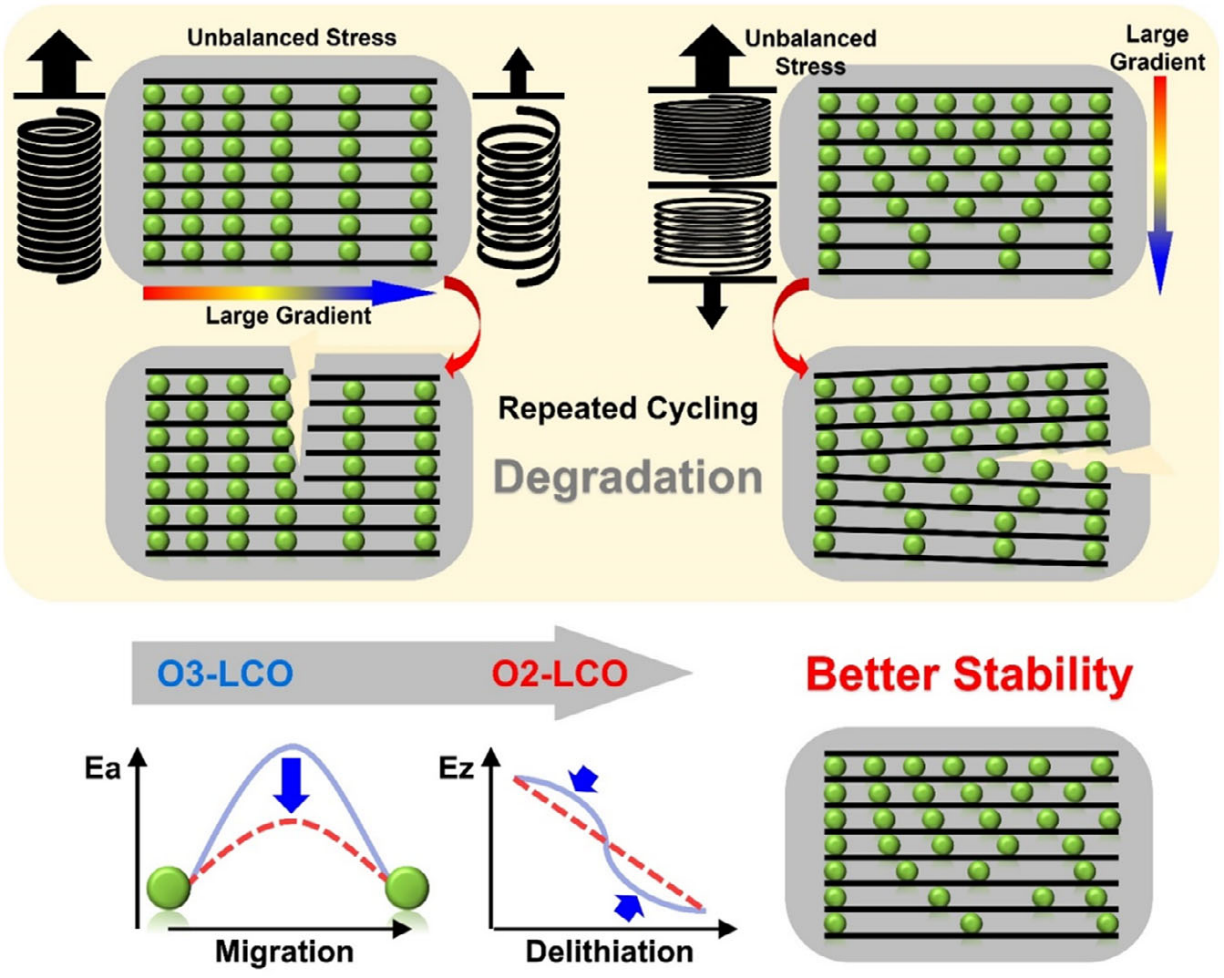
Schematic showing of proposed chemomechanic degradation process for LCO particle during delithiation, and the key factors benefitting O_2_–LCO to achieve stable high‐voltage cycling.

Particle‐level disastrous consequences come with such internal stress building up upon charge–discharge cycling. **Figure** **7** proposes a possible chemomechanical failure mode for an O_3_–LCO particle. Relatively sluggish Li^+^ diffusion induces a large concentration gradient and local strain inside the particle. The nonideal elasticity profile builds up local stress and causes severe imbalance once a local region reaches the Li^+^ concentration threshold. Such repeatedly imbalance occurrence upon cycling would eventually cause crack formation. Imbalance along the slab can tear the slab apart and imbalance directional to the slab can exfoliate the slab. Specifically, for O_3_–LCO, the Li^+^ concentration threshold is around 50% SOC. High‐voltage cycling would inevitably cause all local regions of a particle to pass through this threshold at some point and, therefore, lead to degradation. In O_2_–LCO, Li^+^ diffusion is faster, and the elasticity change is continuous, all of which slows down the internal stress rise and benefits high‐voltage cycling stability.

The more severe particle degradation of O_3_–LCO compared to O_2_–LCO after high‐voltage cycling is also supported by other characterization results in addition to SEM imaging. In Figure S9a–f, Supporting Information, the surface region evolution in LCO particles is monitored by X‐ray photoelectron spectroscopy (XPS) upon high‐voltage cycling. Clearly, decomposition constantly occurs to the O_3_–LCO particle surface as peak intensity keeps growing and new peaks keep emerging, while for O_2_–LCO the XPS peaks barely change after 40 high‐voltage cycles. The results of XRD measurement on cycled LCO sample are displayed in Figure S9g, Supporting Information. O_3_–LCO particles show much worse crystallinity than O_2_–LCO particles, indicated by the very small value of peak intensity ratio *I*_(003)_/*I*_(104)_. Figure S9h, Supporting Information, displays the Raman spectroscopy measured on cycled LCO samples, where peaks corresponding to surface degradation product Co_3_O_4_ are identified for O_3_–LCO but not for O_2_–LCO.

## 3 Conclusion

O_3_– and O_2_–LCO cathode materials were successfully prepared with the identical particle morphology. Careful electrochemical tests reveal that O_2_–LCO exhibits significantly better cycling performance than O_3_–LCO at high cutoff voltages. GITT tests and Li^+^ migration barrier calculations show that O_2_–LCO exhibits superior lithium‐ion diffusion kinetics compared to O_3_–LCO. Further morphological imaging and spectroscopic characterization revealed severe particle‐level degradation of O_3_–LCO after high‐voltage cycling. Multiscale simulation investigated the particle level distribution of Li concentration and stress under the influence of sluggish diffusion and varying elasticity. A chemomechanical failure mechanism is proposed for O_3_–LCO. The fast Li diffusion and ideal elasticity evolution upon delithiation synergistically contribute to the excellent cycling stability of O_2_–LCO with high cutoff voltage.

## 4 Experimental Section

4.1

4.1.1

##### Synthesis of O_2_–LCO and O_3_–LCO

O_2_–LCO was prepared by ion exchanging P_2_–NCO. Stoichiometric Co_3_O_4_ (CNGR Advanced Material Co., Ltd.) and Na_2_CO_3_ (Beijing InnoChem Science & Technology Co., Ltd.) were thoroughly mixed and calcined at 800 °C for 48 h under an oxygen atmosphere to obtain P_2_–NCO. LiCl/LiOH (Beijing InnoChem Science & Technology Co., Ltd.) was dissolved in deionized water at a concentration of 2.5 mol L^−1^, each as the ion‐exchange environment. The P_2_–NCO was added to the solution with a Li/Na molar ration of 10:1. The exchange reaction was performed for 24 h at 100 °C with a reflux condensation. The resulting products from ion exchange were filtered and washed three times, finally yielding O_2_–LCO after drying below 100 °C. O_2_–LCO was further calcined at 600 °C in an oxygen atmosphere for 12 h to obtain O_3_–LCO.

##### Electrochemical Tests

The electrodes were fabricated by mixing LiCoO_2_ powder, carbon black (SUPER C65, Imerys S.A.), and polyvinylidene fluoride (PVDF, Solef 5130, Solvay S.A.) in a mass ratio of 8:1:1. PVDF was dissolved in N‐methyl‐2‐pyrrolidone (TCI [Shanghai] Development Co., Ltd.) in advance at a concentration of 5 wt%. The three substances were mixed in a high‐speed mixer at a rotational speed of 2000 rpm for 15 min to form a homogeneous slurry, and the slurry was then cast on aluminum foil with a blade and dried at 120 °C in a vacuum for 6 h. The electrodes were cut into disks with a diameter of 12 mm and weighted. The mass loading of the active material was about 2–3 mg cm^−^^2^. CR2032‐type (as defined by the International standard of International Electrotechnical Commission 60086‐3) coin cells were assembled in an argon‐filled glove box (O_2_ and H_2_O below 0.1 ppm, MBraun). As‐prepared LiCoO_2_ was used as the cathode and lithium metal foil as the anode. Al_2_O_3_‐coated polymer membrane (Celgard) was used as the separator, and l M LiPF_6_ in ethylene carbonate (EC)/dimethyl carbonate (DEC, 3:7 in volume,) as the electrolyte. The commercial electrolyte with additive (LB‐363) was purchased from Suzhou DoDoChem Science & Technology Co., Ltd. The galvanostatic tests were conducted at a rate of 0.1C (1C = 200 mAh g^−1^) for the initial 2 weeks, followed by a rate of 0.5C, using a Land CT2001A battery test system. The GITT test was performed in a voltage range of 3.0–4.4 V, and 0.05C was applied in each galvanostatic stage (8 min), followed by the rest for 4 h.

##### Characterization

The morphologies of powder samples and electrode samples were detected by an SEM (Hitachi S‐8400). The crystal structure of materials was measured by XRD (Rigaku Smartlab9KW) with Cu Kα radiation. In situ electrochemical XRD was performed using a special model cell in which glass fibers were used as separators. The model cells were charging and discharging at 0.1C in 3–4.7 V. For O_2_–LCO, the initial cycle was conducted at 0.1C, followed by in situ data collection during the second cycle. Raman measurement (HORIBA LabRAM HR Evolution) was performed using 532 nm laser. The surface species of the pristine and cycled electrodes were determined using an X‐ray photoelectron spectrometer (Thermo Fisher ESCALAB 250 Xi) with monochromatic 150 W Al K_α_ radiation

##### DFT Calculation

DFT‐based calculations were carried out to determine the lattice parameter and Young's modulus at different SOC of LCO. All the DFT calculations were performed with the projector‐augmented wave approach,^[^ ^41^, ^42^ ^]^ as implemented in the Vienna Ab initio Simulation Package (VASP). Perdew–Burke–Ernzerhof spin‐polarized generalized gradient approximation (GGA) was adopted for exchange–correlation functional.^[^ ^43^ ^]^ For cobalt, the on‐site Hubbard U correction was applied to correct the self‐interaction errors in GGA, where the effective U–J (U and J stand for the effective on site Coulomb‐ and exchange parameters) values were set as 4.91 eV.^[^ ^44^ ^]^ The plane wave cutoff energy of 500 eV and the k‐mesh density of on point per ≈0.03 Å^−3^ were employed. For structural relaxation, the lattice parameters and atomic positions were fully relaxed until the forces felt by each atom were less than 0.001 eV Å^−1^. The van‐der‐Waals‐modified DFT‐D3 method with Becke–Johnson damping was employed to address the weak interaction between adjacent CoO_6_ slabs in delithiated states. This method has been reported to offer relatively accurate description of inter‐slab distance at a reasonable cost of computational resources.^[^ ^33^ ^]^ Supercell model was constructed for O_3_–Li_*x*_CoO_2_ at *x* = 1, 0.857, 0.67, 0.5, 0.33, 0.25, 0.168, 0 and for O_2_–Li_*x*_CoO_2_ at *x* = 1, 0.75, 0.67, 0.5, 0.33, 0.25, 0. Structural illustrations of supercell models used in current work are shown in Figure S10 and S11, Supporting Information. The slab shifting and in‐plane Li‐vacancy configuration were determined following previous literatures.^[^ ^30^, ^32^, ^33^ ^]^ Stress–strain method as implemented in VASP was used to calculate the Young's modulus. The resultant elastic modulus matrix is represented following the voigt labeling as follows:

(1) $$\left(\begin{array}{cccccc} C_{11} & C_{12} & C_{13} & 0 & 0 & 0\\ C_{21} & C_{22} & C_{23} & 0 & 0 & 0\\ C_{31} & C_{32} & C_{33} & 0 & 0 & 0\\ 0 & 0 & 0 & C_{44} & 0 & 0\\ 0 & 0 & 0 & 0 & C_{44} & 0\\ 0 & 0 & 0 & 0 & 0 & C_{66} \end{array}\right)$$

The corresponding meanings of each individual element of the matrix can be found in the literature.^[^ ^45^ ^]^

##### FEM Simulation

This simulation focused on the nonuniformity of the electrochemical process and coupled particle stress in a half‐cell model with a plate‐like cylindrical LiCoO_2_ particle. Detailed model sizes are displayed in Figure S12, Supporting Information. A representative volume element was constructed to contain the statistical features of the macroscopic particle ensembles. First, we outlined the theoretical framework of the coupled electrochemo‐mechanics model, which shows the heterogeneity in battery. Second, we conducted the electrochemical simulation and obtained the heterogeneous process of Li‐ion transports during the operation of battery. Finally, we proceeded to model the anisotropic stress of an active LiCoO_2_ particle coupled with electrochemical process. We evaluate the impact of anisotropic parameters on both the electrochemical process and the mechanics change in the operating batteries. All model construction and FEM simulation were done with COMSOL 6.1. The half‐cell model consisted of a separator and an LiCoO_2_ single‐particle cathode soaked in 1:1 EC:DEC with 1M LiPF6 liquid electrolyte, a Li anode, and a current collector on the cathode side. The *c* axis of LCO crystal was set as the *z* direction of the cylinder in the geometry. To avoid singularity and increase accuracy of our simulation, a chamfer was added to the cylinder. Then, fully coupled electrochemical‐dilute fluid transport‐solid mechanics interfaces were added to describe the process of electrochemistry in the battery. The charge–discharge rate was set to be 0.5C, minimum voltage was 3 V, maximum voltage was 4.6 V, and the resting time was 360 s. The diffusion coefficients along *x*–*y* axis were set to 2.05 × 10^−14^ m^2^ s^−1^ for O_3_–LCO and 4.91 × 10^−14^ m^2^ s^−1^ for O_2_–LCO based on the GITT measurements. The diffusion coefficient along *z* axis was set to be two orders of magnitude smaller than that along *x* and *y* axis to represent the anisotropic Li ion diffusion. The equilibrium potential curves corresponding to O_3_– and O_2_–LCO were implemented based on the measured voltage profile. The strain and elastic modulus matrices were parameterized as piecewise function based on DFT calculation results (Figure S13 and 14, Supporting Information). The bottom surface of the cylinder was meshed by “mapping” and the mesh of the whole cylinder was determined with an eight‐layer sweep of the mesh on the bottom surface. The rest of the geometry was meshed by traditional tetrahedron at ultrafine level.

## Conflict of Interest

The authors declare no conflict of interest.

## Supporting information

Supplementary Material

## Data Availability

The data that support the findings of this study are available from the corresponding author upon reasonable request.
