# Supplementary material for: On the Much‐Improved High‐Voltage Cycling Performance of LiCoO2 by Phase Alteration from O3 to O2 Structure
Source: Small Sci. 2024 Aug 1;4(10):2400162. doi: 10.1002/smsc.202400162 (PMC11935130; doi:10.1002/smsc.202400162)
Supplement: Supplementary file 1 — Supplementary Material [file SMSC-4-2400162-s001.pdf]

Supporting Information

On the much-improved high voltage cycling performance of $LiCoO_2$ by phase alteration from O3 to O2 structure

Mingwei Zan, Hongsheng Xie, Sichen Jiao, Kai Jiang, Xuelong Wang*, Ruijuan Xiao*, Xiqian Yu*, Hong Li, and Xuejie Huang

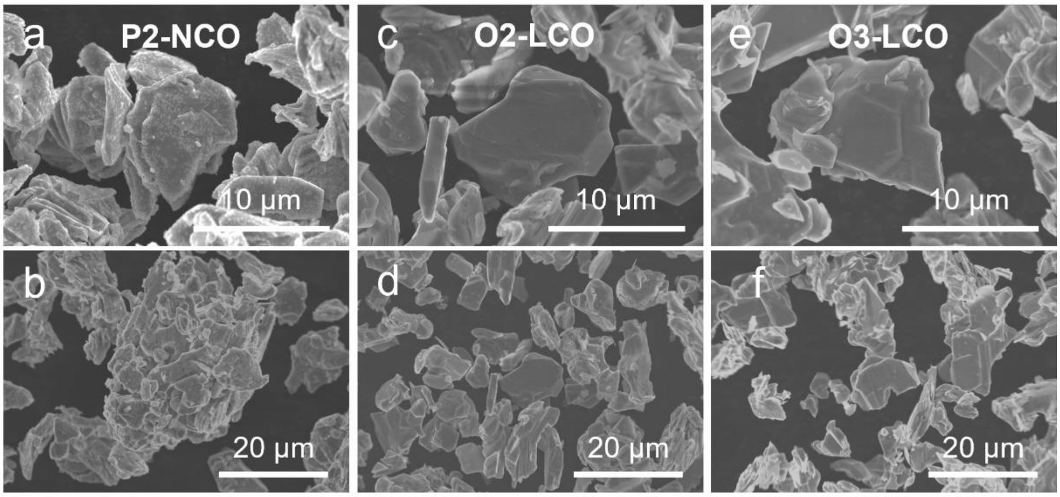

Figure S1. SEM images of (a)-(b) as-synthesized P2-NCO, (c)-(d) as-synthesized O2-LCO, and (e)-(f) as-synthesized O3-LCO.

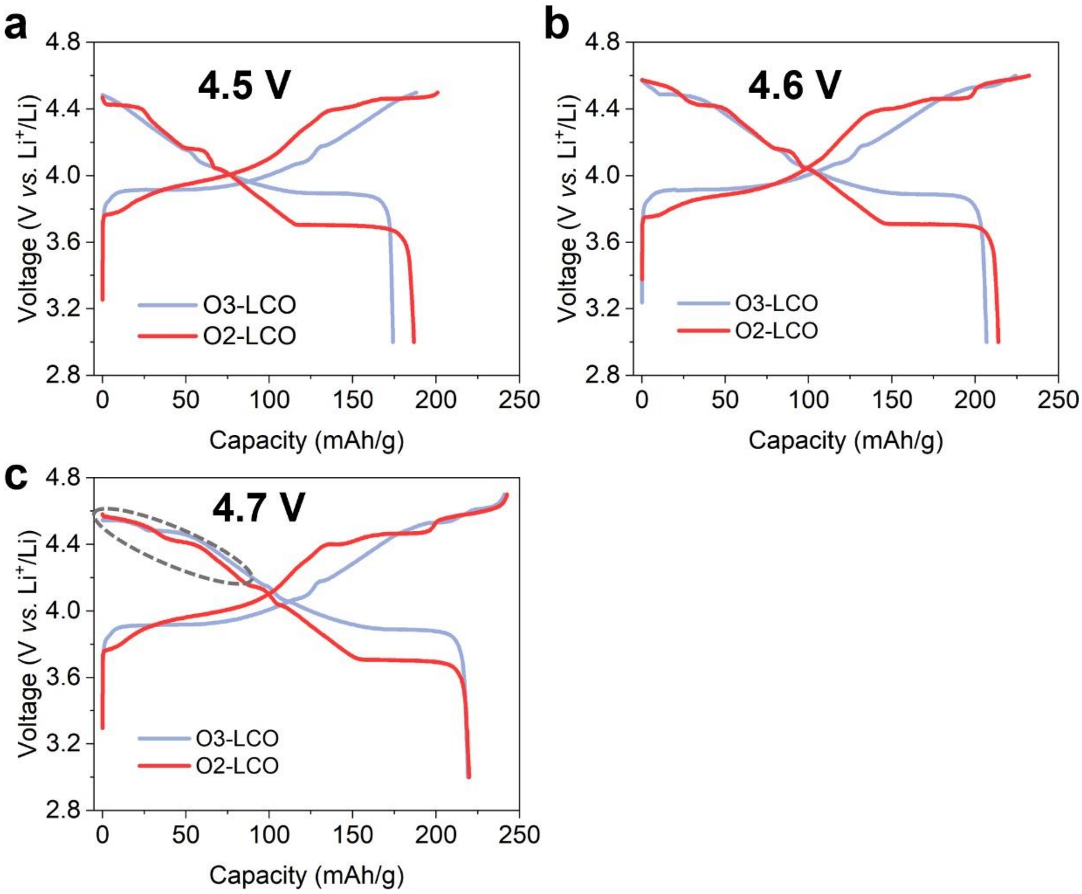

Figure S2. 1st cycle voltage profile comparison between O3 and O2-LCO between 3V and (a) 4.5 V, (b) 4.6 V, and (c) 4.7 V vs. Li^{+}/Li.

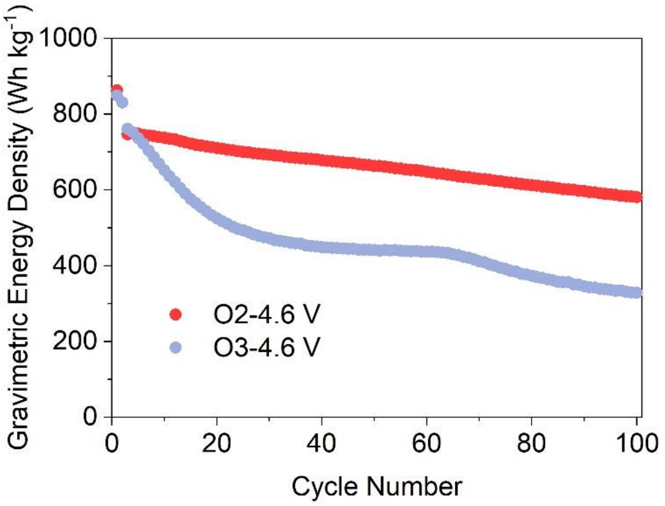

Figure S3. Cycling energy density comparison between O3 and O2 $LiCoO_2$ between 3V and 4.6 V vs. Li^+/Li.

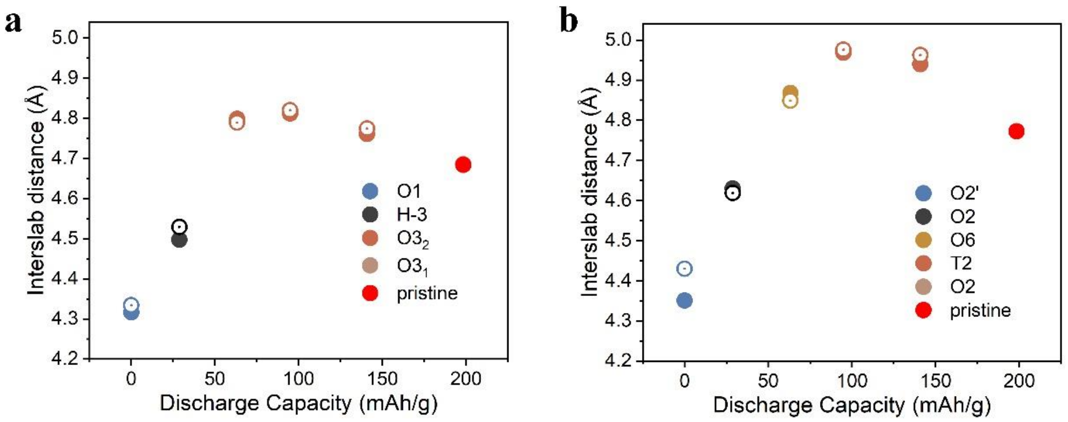

Figure S4. The interslab distance change during discharge process in representative intermediate phases, obtained from in-situ XRD measurement for (a) O3 and (b) O2 $LiCoO_2$. Data are compared with the one during charge process (hollow points)

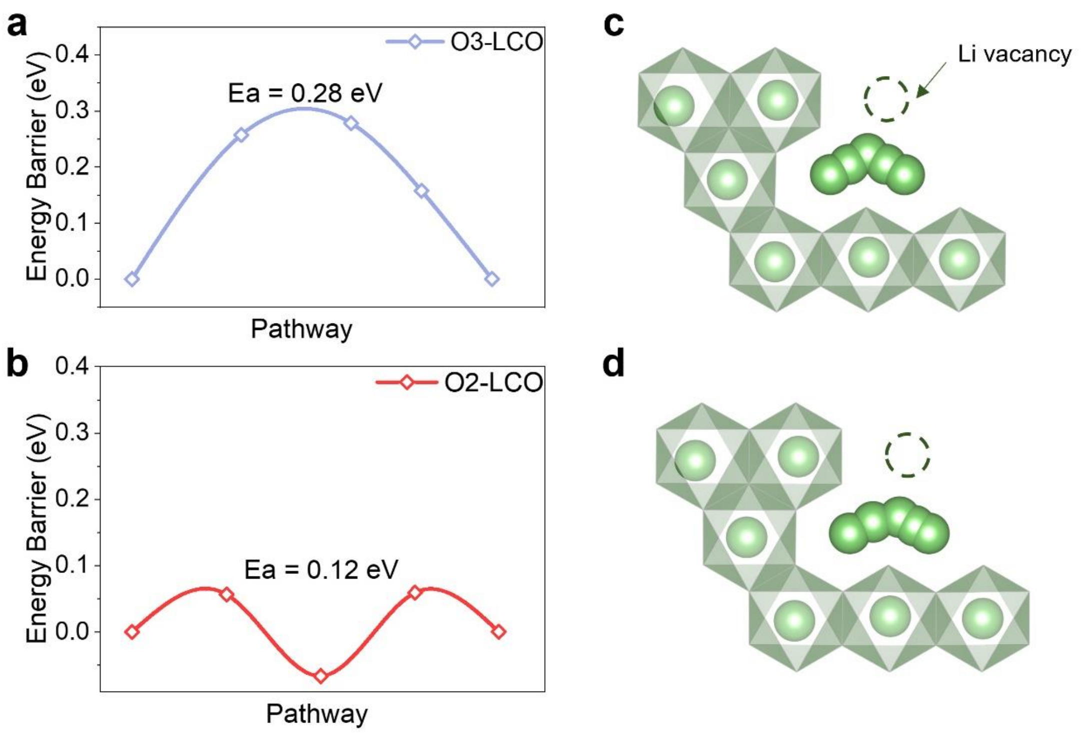

Figure S5. Energy pathway for di-vacancy Li+ migration in (a) O3-LCO and (b) O2-LCO. Trajectory of corresponding Li^+ migration viewed along c-axis for (c) O3-LCO and (d) O2-LCO.

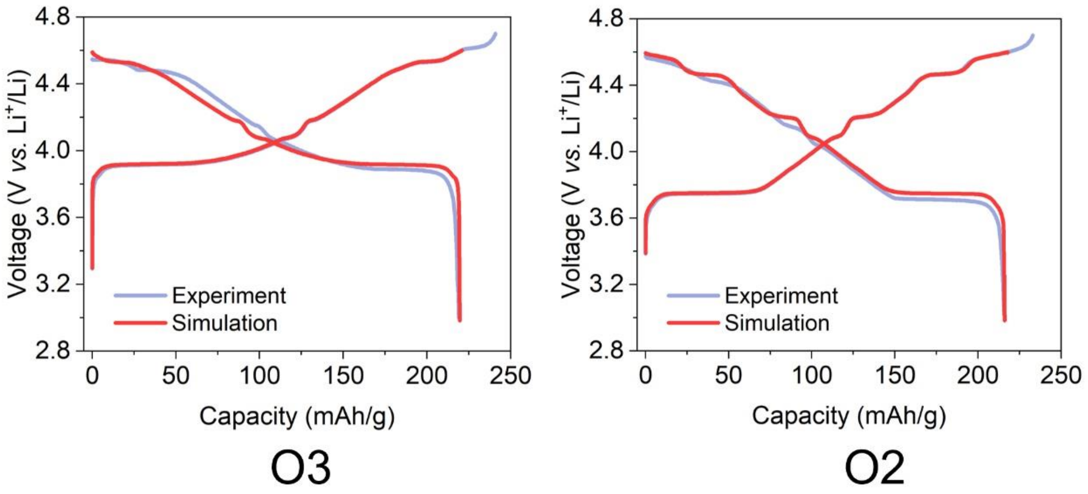

Figure S6. Voltage profile comparison between measured and simulated for O3-LCO (left) and O2-LCO (right).

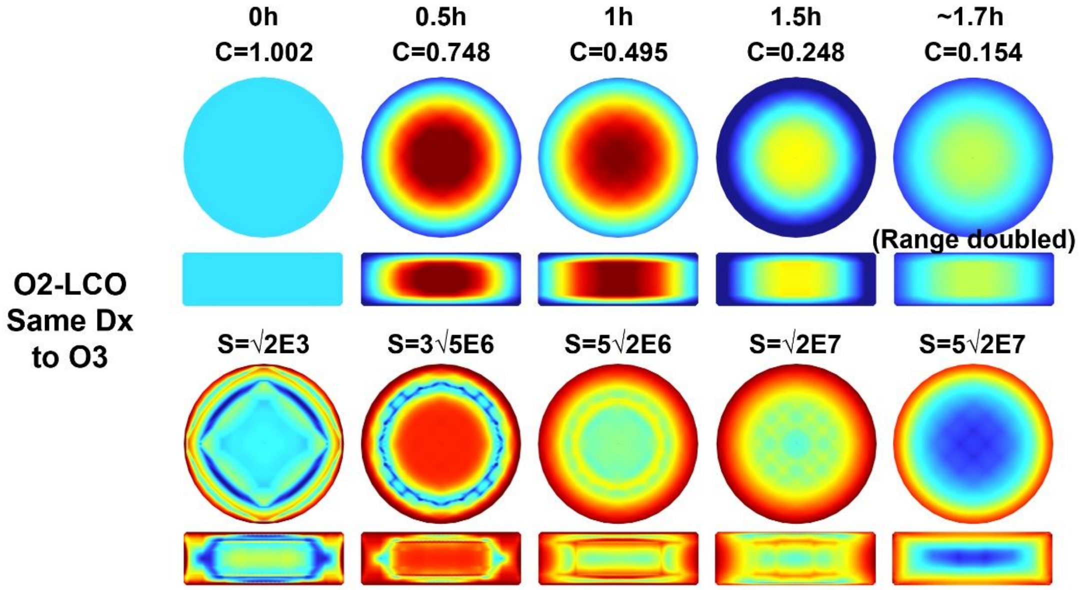

Figure S7. Distribution of Li^+ concentration (upper) and internal stress (lower) at different SOC simulated with diffusivity of Li^+ in O2-LCO changed to the value in O3-LCO.

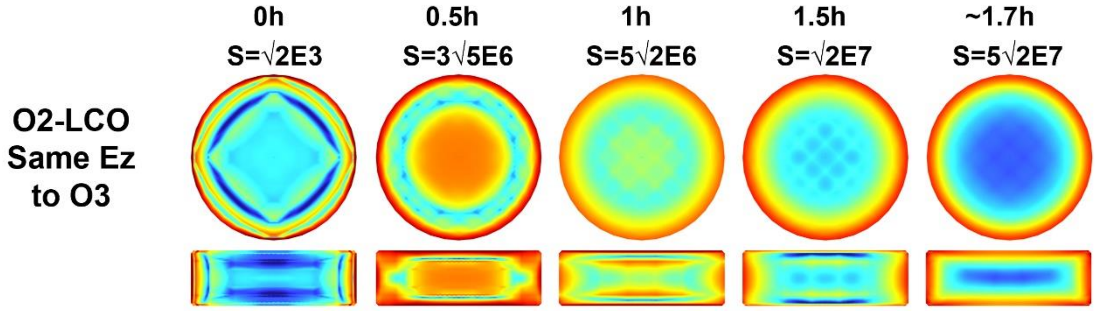

Figure S8. Distribution of internal stress at different SOC simulated with elastic moduli along z direction in O2-LCO changed to the value in O3-LCO.

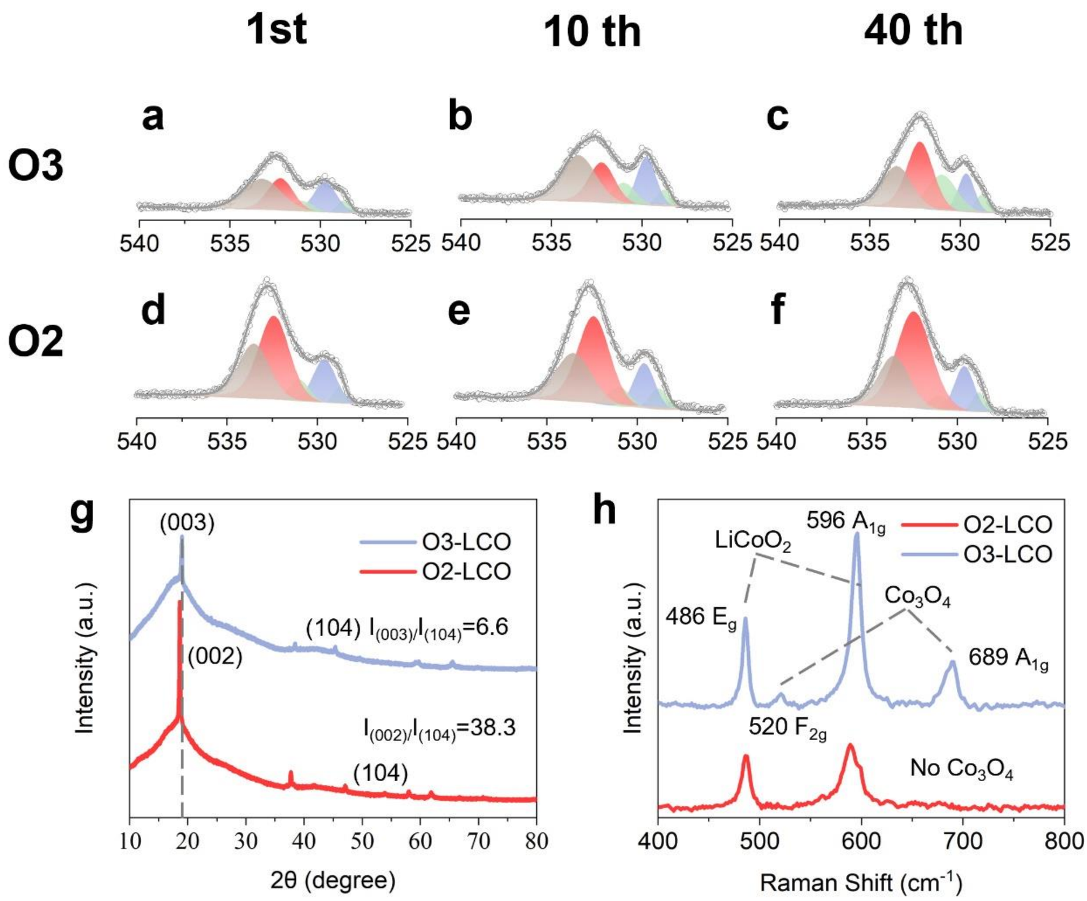

Figure S9. XPS results on (a)-(c) O3-LCO sample and (d)-(f) O2-LCO sample after 1st, 10th, and 40th cycle, respectively. (g) XRD and (h) Raman results comparison between O3 and O2-LCO sample after 100 high-voltage cycling.

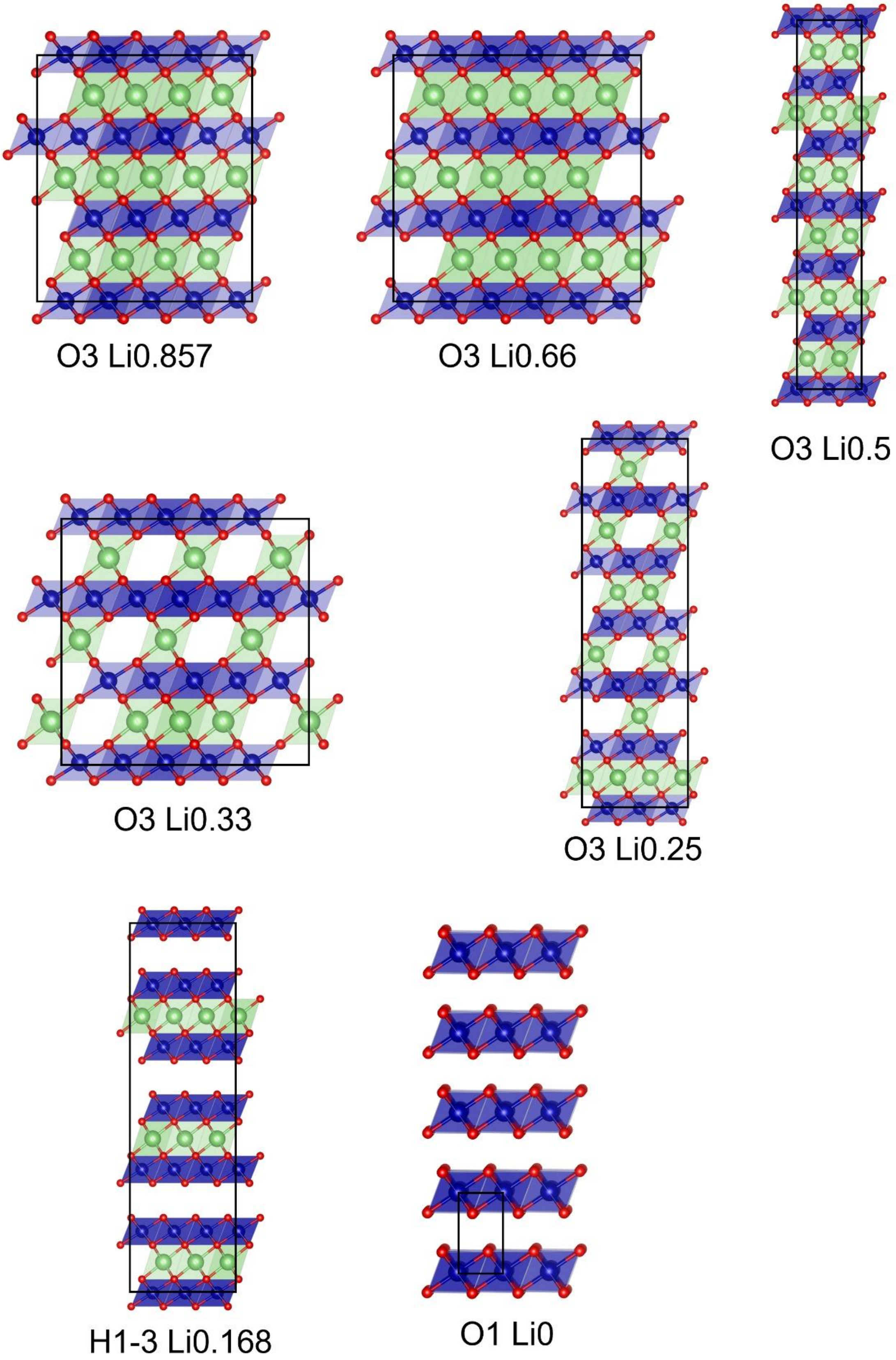

Figure S10. Illustrations of supercell used in DFT calculation for O3-LCO at different delithiated states.

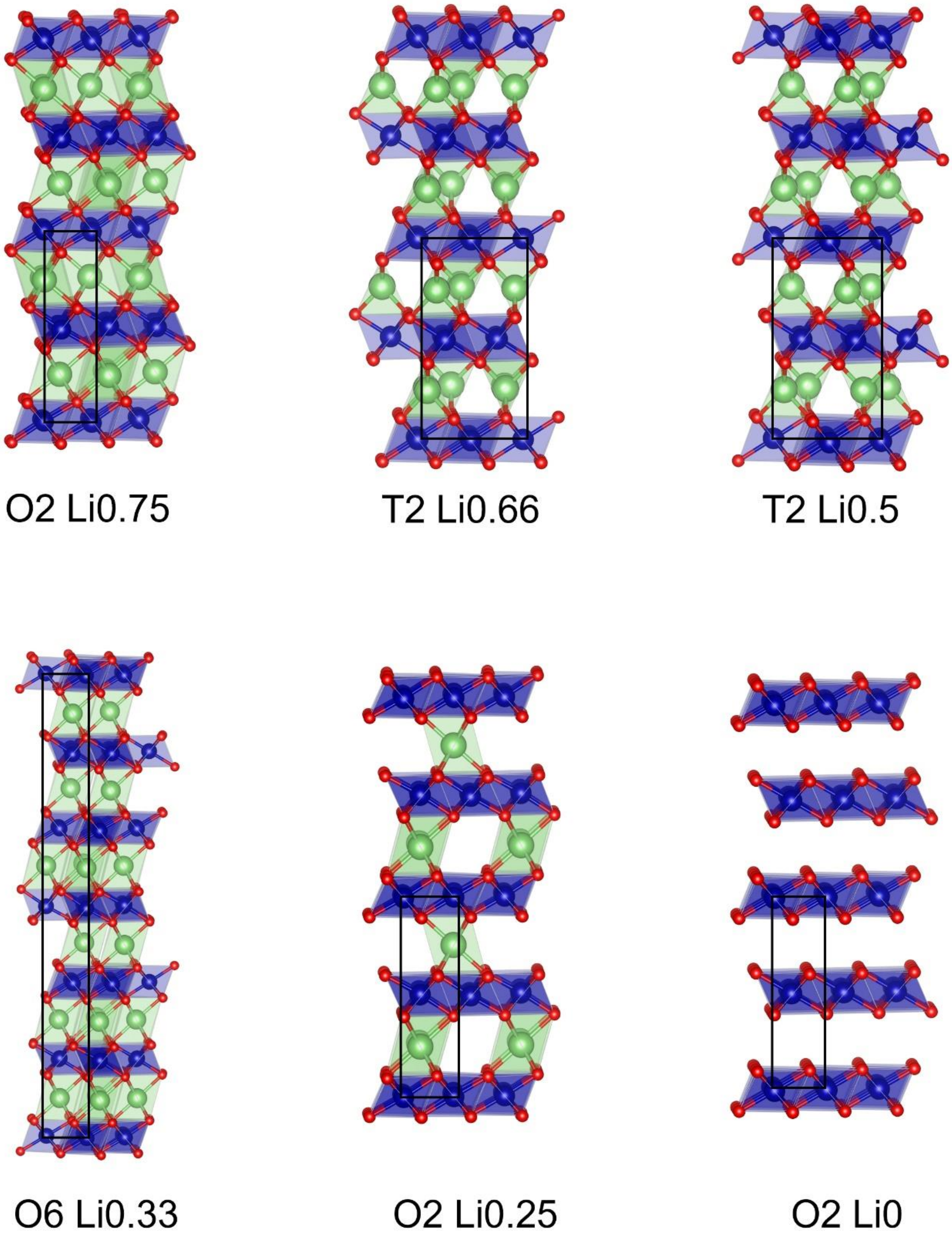

Figure S11. Illustrations of supercell used in DFT calculation for O2-LCO at different delithiated states.

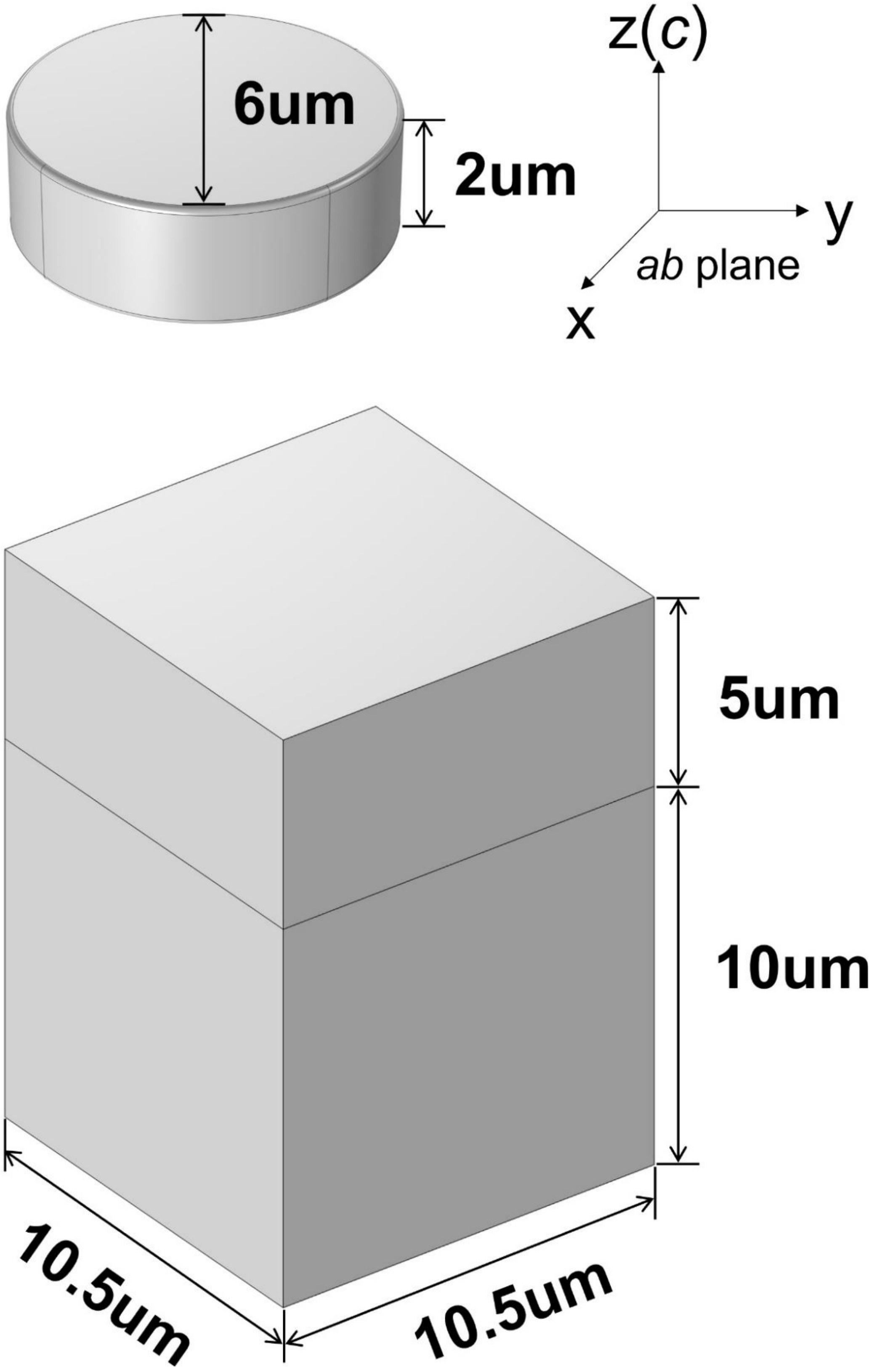

Figure S12. Illustrations of single particle cell model used in FEM simulation with COMSOL.

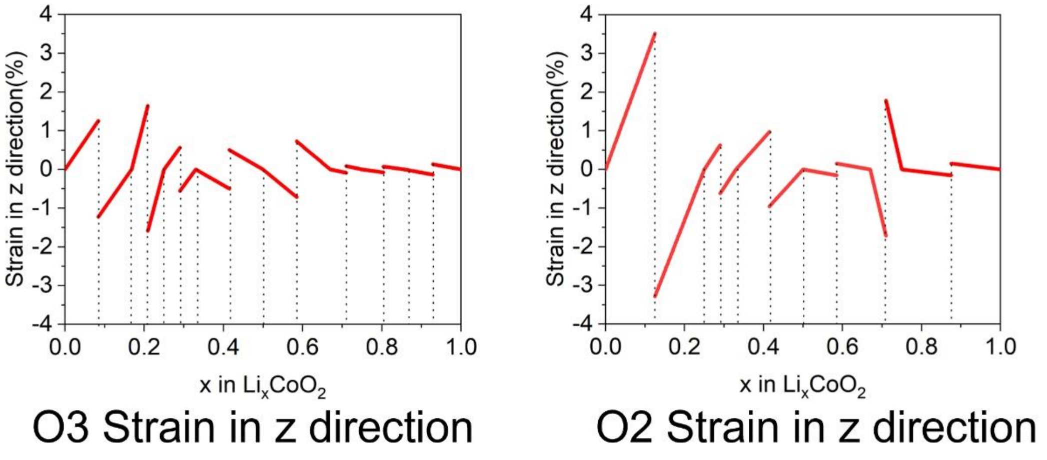

Figure S13. Piecewise function used to parameterize strain evolution upon delithiation in FEM simulation based on DFT results.

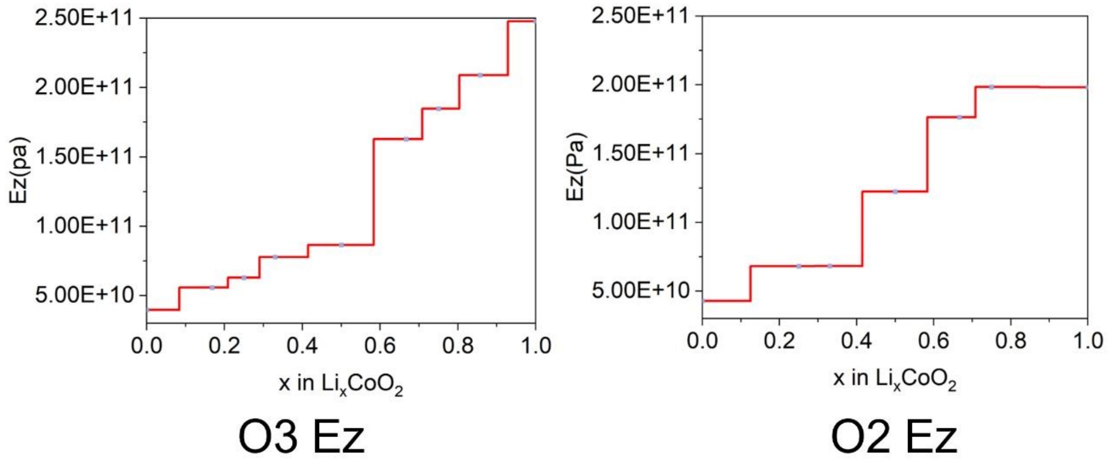

Figure S14. Piecewise function used to parameterize elastic moduli evolution upon delithiation in FEM simulation based on DFT results. Only E_z is displayed as representative. Other elements of Young's Modulus matrix were parameterized in the same fashion.

Table S1. DFT calculated lattice parameter change for O3-LCO at different delithiated states.

x	Host	a (Å)	c (Å)
0.00	O1	2.82	4.47
0.167	H1-3	2.83	4.58
0.25	O3	2.80	4.73
0.33	O3	2.79	4.78
0.50	O3	2.79	4.74
0.67	O3	2.79	4.67
0.75	O3	2.80	4.66
0.857	O3	2.80	4.65
1.00	O3	2.80	4.64

Table S2. DFT calculated lattice parameter change for O2-LCO at different delithiated states.

x	Host	a (Å)	c (Å)
0.00	O2	2.82	4.45
0.25	O2	2.80	4.77
0.33	O6	2.81	4.83
0.50	T2	2.80	4.92
0.67	T2	2.80	4.89
0.75	O2	2.79	4.74
1.00	O2	2.79	4.72

Table S3. DFT calculated elastic moduli change for O3-LCO at different delithiated states.

x	Ex/y (GPa)	Ez (GPa)
0.00	269.00	39.80
0.167	255.40	55.80
0.25	296.40	62.90
0.33	294.60	77.80
0.50	320.40	86.50
0.67	350.20	162.80
0.75	351.20	184.80
0.857	360.30	208.90
1.00	377.30	247.80

Table S4. DFT calculated elastic moduli change for O2-LCO at different delithiated states.

x	Ex/y (GPa)	Ez (GPa)
0.00	268.69	43.05
0.25	286.72	67.97
0.33	268.96	68.22
0.50	304.32	122.32
0.67	315.84	160.78
0.75	345.35	198.38
1.00	345.45	198.18
